# Diabetic dry eye: advances in pathogenesis, diagnostic strategies, and therapeutic approaches

**DOI:** 10.3389/fmed.2026.1756568

**Published:** 2026-02-05

**Authors:** Ting Huang, Dahu Wang, Dan Jiang, Xuejing Lu, Lan Lin, Yuyan Zhang, Xin Li, Yihong Hou, Hong Li, Xinquan Liu

**Affiliations:** 1 Department of Ophthalmology, Longhua Hospital Shanghai University of Traditional Chinese Medicine, Shanghai, China; 2 Eye School, Chengdu University of Traditional Chinese Medicine, Chengdu, China; 3 Department of Ophthalmology, The Second Affiliated Hospital of Fujian University of Traditional Chinese Medicine, Fuzhou, China; 4 Department of Endocrinology, Longhua Hospital Shanghai University of Traditional Chinese Medicine, Shanghai, China

**Keywords:** diabetic dry eye, corneal neuropathy, tear film instability, confocal microscopy, tear fluid biomarkers, therapeutic interventions

## Abstract

Dry eye is the most common ocular surface disorder that is increasingly acknowledged to be associated with diabetes mellitus. Via metabolic dysregulation and neural injury, diabetes mellitus significantly increases the prevalence of dry eye, adversely affecting patients’ quality of life. At present, the diagnosis and treatment of diabetic dry eye are still facing challenges in clinical practice. This article outlines the prevalence and risk factors associated with diabetic dry eye, explores its underlying pathogenic mechanisms, such as advanced glycation end-product accumulation, oxidative stress, corneal neuropathy, and impaired neural regulation, which collectively disrupt the lacrimal functional unit, leading to reduced tear secretion and tear film instability. The clinical manifestations of diabetic dry eye are also reviewed. According to current literature, diagnostic strategies utilizing confocal microscopy and tear fluid biomarkers are proposed. In addition, this review summarizes recent therapeutic advances and potential intervention strategies for diabetic dry eye, with a focus on emerging mechanism-based treatments. Taken together, this review aims to advance research on diabetic dry eye and offer novel insights to support early diagnosis and precision therapy.

## Graphical abstract

**Figure:**
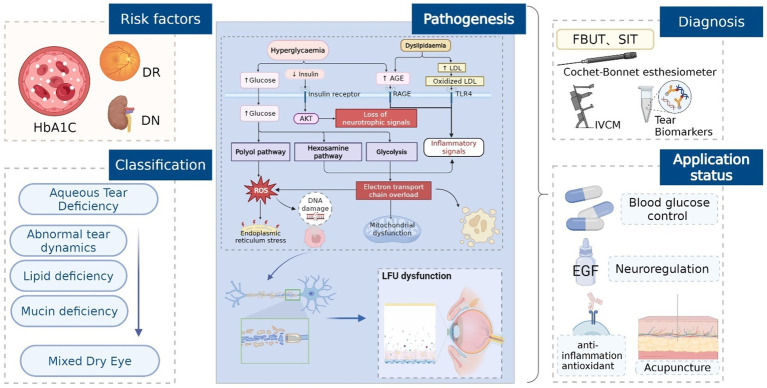
Created in BioRender. Ting, H. (2026) https://BioRender.com/lv1m7hg.

## 1 Introduction

Diabetes mellitus (DM) is a prevalent metabolic disorder with substantial implications for global public health. According to the International Diabetes Federation, the global prevalence of DM among adults aged 20–79 reached 10.5% (536.6 million individuals) in 2021 (1). This figure is projected to rise by approximately 2.5% annually (2), reaching 783.2 million by 2050 (1, 3). Beyond its systemic complications, DM has a profound impact on ocular health, notably contributing to an increased prevalence of dry eye (4, 5). Dry eye is a prevalent ocular surface condition characterized by symptoms such as dryness, foreign body sensation, burning, and fluctuating vision, which can significantly impair quality of life (6). Multiple studies have reported that individuals with DM exhibit a markedly higher prevalence of dry eye, with strong correlations to disease duration and glycemic control (7, 8).

Increasingly recognized as a distinct clinical entity, diabetic dry eye is characterized by complex pathogenesis and heterogeneous symptoms but lacks standardized diagnostic criteria. Current treatments remain predominantly empirical and fail to target the specific pathophysiology of the disease, thereby impeding timely diagnosis and effective management in clinical practice. The pathogenesis of dry eye involves interrelated processes such as tear hyposecretion, tear film instability, and ocular surface inflammation. In individuals with DM, metabolic dysregulation may lead to lacrimal gland dysfunction, corneal nerve damage, and pathological alterations in ocular surface cells, thereby collectively elevating the risk of dry eye syndrome (9, 10). Moreover, DM can exacerbate ocular surface disease by inducing chronic inflammation (11). Recent research has increasingly emphasized marked differences in the clinical manifestations and pathological mechanisms of dry eye between diabetic and non-diabetic populations (12, 13). These findings establish a theoretical framework for the development of targeted management strategies for diabetic dry eye.

Consequently, a comprehensive synthesis of the epidemiology, classification, pathogenesis, diagnostic advances, and therapeutic progress in diabetic dry eye is essential for guiding clinical practice and promoting further research. This review aims to provide clinicians and researchers with an integrated reference to support precision diagnosis and treatment, thereby improving ocular surface health and quality of life in affected individuals.

## 2 Epidemiology

DM has been identified as a major systemic risk factor for dry eye (14). Epidemiological data indicate a wide global prevalence of dry eye, ranging from 5 to 50% (15). DM increases the risk of developing dry eye by 2- to 3-fold, with prevalence rates exceeding 50% in diabetic populations (16–18). Several studies have shown associations between the occurrence of diabetic dry eye and various factors, such as patient age, blood glucose levels, glycated hemoglobin (HbA1c), and the duration of DM (7, 18, 19). However, the available evidence is not entirely consistent. A meta-analysis reported that elderly patients with diabetes exhibit better tear function than younger patients (23). While other studies have not observed a significant association between diabetes duration or random blood glucose levels and dry eye (20, 21).

In this context, accumulating evidence suggests that long-term glycemic control may be a key determinant of ocular surface health. Derakhshan et al. proposed that poor glycemic control is an important factor contributing to ocular surface damage in patients with diabetes (22). Consistent with this, a meta-analysis by Kuo et al. demonstrated that diabetic patients with well-controlled glycemia exhibit tear function comparable to that of non-diabetic individuals (23). Based on these findings, HbA1c is considered a relatively stable and independent risk factor for diabetic dry eye (19), with higher HbA1c levels being associated with an increased risk of the condition (24). Consequently, dry eye assessments ought to be systematically integrated into ocular examinations for patients exhibiting poor glycemic control.

Beyond glycemic control, diabetic dry eye is also closely associated with multiple microvascular complications. Previous studies have shown that the prevalence of dry eye is significantly increased in patients with diabetic retinopathy (DR) and diabetic nephropathy (DN). A cross-sectional study involving 105 patients with type 2 diabetes mellitus reported an overall dry eye prevalence of 43.81%, and notably, 61.96% of patients with dry eye were found to have proliferative diabetic retinopathy (PDR), suggesting that the presence of dry eye may be associated with the severity of DR (24). Tran et al. (19) reported a significantly higher prevalence of dry eye among patients with DN compared with those without renal complications, and a reduced estimated glomerular filtration rate (eGFR) was identified as an independent risk factor for the development of dry eye. In addition to shared metabolic and inflammatory mechanisms, the accumulation of systemic metabolic products and toxins secondary to renal dysfunction may further disrupt ocular surface homeostasis and promote the development of dry eye (25, 26). These findings suggest that ocular surface alterations associated with diabetes may precede the development of other microvascular complications in some patients, offering potential insights for the early detection of diabetic complications.

## 3 Pathogenesis

### 3.1 Mechanisms underlying hyperglycemia-induced ocular surface damage

Metabolic dysfunction associated with diabetes can lead to degenerative alterations in ocular vasculature and neural structures, thereby promoting the onset and progression of dry eye (27). These processes are largely driven by metabolic imbalance, oxidative stress, and chronic inflammation (28); a clearer understanding of their interrelationships is essential for early recognition and optimal management of diabetic dry eye. As a central driving factor, sustained hyperglycemia promotes the diversion of excess glucose from glycolysis into the polyol and hexosamine pathways, thereby markedly exacerbating oxidative stress and facilitating the abnormal accumulation of advanced glycation end products (AGEs) (10, 29, 30). These metabolic abnormalities activate pattern recognition receptors, such as the receptor for AGEs (RAGE) and Toll-like receptor 4 (TLR4), subsequently triggering downstream inflammatory signaling pathways, such as NF-κB, MAPK, and JAK/STAT. The activation of these pathways results in aberrant expression of pro-inflammatory cytokines and matrix metalloproteinases, disrupts corneal nerve homeostasis, and induces apoptosis and functional impairment of corneal epithelial cells, lacrimal glands, and other ocular surface-related cells (31–34), thereby driving the initiation and progression of diabetic dry eye.

A systematic review encompassing 30 studies with a total of 871 participants identified a range of tear fluid biomarkers that may reflect diabetes-related ocular surface alterations. These included cytokines (IL-6, IL-8, TNF-*α*, and MMP-9), neuropeptides (substance P and neuropeptide Y), proteins (IGFBP-3 and progranulin), as well as lipids, glycans, microRNAs, circular RNAs, and trace elements (35). *In vivo* confocal microscopy (IVCM) studies have further demonstrated a significantly increased density of dendritic cells (DCs) in the corneas of patients with diabetic retinopathy, accompanied by impaired corneal nerve parameters and aggravated dry eye symptoms (36). As key antigen-presenting cells, activated DCs recruit CD4^+^ T cells, particularly Th1 and Th17 subsets, which secrete interferon-γ (IFN-γ), interleukin-17 (IL-17), IL-1β, and IL-6 into the tear film. This process leads to increased tear osmolarity and further amplification of local inflammatory responses (37–39). Concurrently, hyperglycemia-induced inflammation and oxidative stress can sustain the activation of matrix metalloproteinases (MMPs), thereby promoting the degradation of corneal epithelial components and tight junction proteins and ultimately compromising the ocular surface barrier. Progranulin (PGRN) is a glycoprotein that plays an important role in maintaining ocular surface homeostasis through its anti-inflammatory and tissue-protective properties (40, 41). Zhou et al. compared tear PGRN levels between patients with type 2 diabetes mellitus and healthy controls and found that PGRN concentrations were significantly reduced in diabetic patients. Moreover, tear PGRN levels were positively correlated with corneal nerve parameters, tear breakup time (TBUT), and Schirmer test scores (36), suggesting that reduced PGRN may weaken endogenous negative feedback regulation of ocular surface inflammation.

In the setting of persistent metabolic dysregulation and chronic inflammation, corneal nerves are among the earliest structures to be affected (42). Given their essential role in corneal sensation, reflex tear secretion, and ocular surface homeostasis, clarification of the mechanisms driving corneal neuropathy in diabetes is fundamental to understanding the pathological basis of diabetic ocular disease.

### 3.2 Corneal neuropathy

Corneal nerve axon terminals are enriched with mitochondria and exhibit high metabolic demands, rendering neurons particularly susceptible to oxidative stress–induced injury (43). Reactive oxygen species (ROS) can directly damage neuronal membrane lipids, mitochondrial DNA, and axonal transport–related proteins, thereby triggering axonal degeneration and neuronal apoptosis. Compared with non-diabetic individuals, patients with early diabetic peripheral neuropathy exhibit significant reductions in corneal nerve fiber density (CNFD), corneal nerve branch density (CNBD), and corneal nerve fiber length (CNFL) (44–46). IVCM has further revealed the diminished “beaded” morphology of corneal nerve fibers in diabetic patients, which is indicative of nerve damage or degeneration. This reduction suggests impaired metabolic function and degenerative changes in the corneal nerves (47).

Corneal nerves are predominantly sensory nerves, primarily responsible for perceiving external stimuli and maintaining tear film distribution and ocular surface homeostasis through the regulation of the blink reflex. Mvilongo et al. (48) assessed corneal sensitivity using the Cochet–Bonnet esthesiometer and found significantly reduced sensitivity in diabetic patients (44.56 ± 9.59 mm) compared with non-diabetic controls (53.59 ± 6.30 mm). Similar results have been reported elsewhere (49). Corneal hypoesthesia leads to reduced blink frequency and amplitude, thereby compromising ocular surface protection and lubrication. As corneal nerve damage progresses, sensory function further declines, giving rise to a clinical phenotype characterized by prominent objective corneal signs in the presence of relatively mild subjective symptoms. This symptom–sign discordance, which is frequently observed in diabetic dry eye, may delay disease recognition and timely intervention (50, 51). Beyond sensory transduction, corneal sensory nerves release neuroregulatory mediators, such as substance P (SP) and calcitonin gene-related peptide (CGRP), which promote corneal epithelial cell migration and proliferation, enhance tight junction integrity, and suppress local inflammatory responses, thereby preserving ocular surface barrier function. Under diabetic conditions, corneal nerve degeneration and reduced neuropeptide secretion may impair neuroepithelial interactions, clinically manifesting as persistent punctate epithelial erosions (52–54).

Meanwhile, the autonomic nervous system innervating the lacrimal glands, meibomian glands, and goblet cells also plays a critical role in maintaining ocular surface homeostasis (55–58). Parasympathetic nerve terminals innervating the lacrimal gland release neurotransmitters such as acetylcholine (ACh), vasoactive intestinal peptide (VIP), and norepinephrine (NE), which bind to cholinergic, VIP, and α1-adrenergic receptors on acinar and ductal epithelial cells. This interaction stimulates the secretion of water, electrolytes, and proteins (55, 59). In diabetic models, increased sympathetic innervation and decreased parasympathetic activity result in excessive NE release, activation of *α*1-adrenergic receptors, mitochondrial dysfunction, and reduced tear production (60, 61). Studies have demonstrated that pharmacological inhibition of sympathetic neural pathways or blockade of α₁-adrenergic receptors can effectively alleviate ocular surface damage and improve tear secretion (62). Consequently, restoration of lacrimal gland neural regulation and preservation of the structural and functional integrity of corneal sensory nerves are of critical importance in the management of diabetic dry eye (63).

### 3.3 Lacrimal functional unit dysfunction

**Figure 1:**
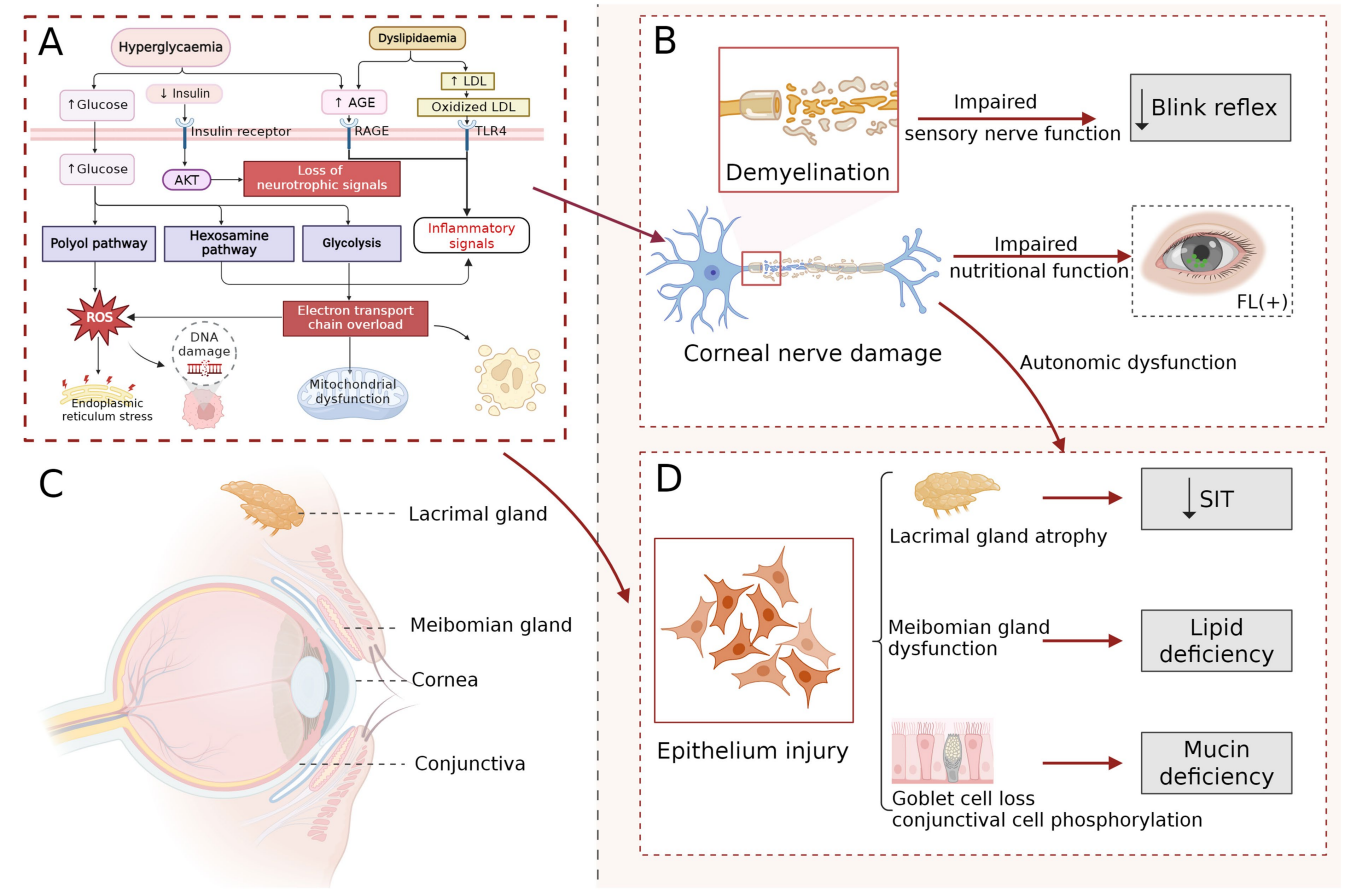
Pathogenesis of diabetic dry eye. Molecular mechanisms primarily encompass damage to corneal nerves and epithelial cells mediated by glycolytic pathway dysregulation. Metabolic disturbances induce mitochondrial dysfunction via the accumulation of AGEs, activation of the polyol pathway, and oxidative stress, thereby eliciting ocular surface inflammation and apoptosis **(A)**. Diabetic nerve fiber injury results in demyelination and neuronal apoptosis, clinically manifesting as decreased corneal sensitivity and impaired blink reflex. Corneal nerve dysfunction reduces trophic support to epithelial cells, leading to persistent corneal fluorescein staining. Autonomic nerve impairment further disrupts ocular surface innervation, thereby exacerbating the dry eye vicious cycle **(B)**. Illustration of the normal ocular surface anatomy **(C)**. Diabetic injury decreases tear secretion from the lacrimal gland, induces damage to the meibomian glands, leading to lipid deficiency, and promotes conjunctival epithelial phosphorylation along with goblet cell loss, thereby resulting in mucin deficiency. Collectively, these alterations contribute to the development of diabetic dry eye **(D)**. Created in BioRender. Ting, H. (2026) https://BioRender.com/kwlpdzg.

The lacrimal functional unit (LFU) consists of the cornea, conjunctiva, lacrimal glands, eyelids, and their associated neural pathways, which together coordinate the production, distribution, and stability of the tear film. Under diabetic conditions, sustained hyperglycemia can markedly disrupt both the structural integrity and regulatory function of the LFU, leading to tear film homeostatic imbalance. In multiple diabetic animal models, diabetic rodents have been shown to exhibit a significant reduction in lacrimal gland weight, accompanied by abnormal glandular differentiation and increased cellular apoptosis, along with a marked decrease in conjunctival goblet cell density on the ocular surface (4, 64–66). Further studies have shown that early ocular alterations in diabetic mice predominantly involve the lacrimal glands, followed by dysfunction of conjunctival goblet cells and subsequent morphological changes in the cornea (67). In addition, non-invasive meibomian gland imaging has revealed varying degrees of gland dropout and atrophy in patients with diabetes, with severity closely associated with longer disease duration and poor metabolic control (68, 69). Together with the aforementioned corneal neuropathy and neural regulatory abnormalities, these findings indicate that LFU dysfunction plays a central role in the development and progression of diabetic dry eye. Accordingly, characterization of structural and functional alterations across LFU components may facilitate interpretation of disease phenotypes and guide targeted interventions (Figure 1).

### 3.4 Decreased tear secretion and altered tear composition

The tear film, composed of mucin, aqueous, and lipid layers, also contains antimicrobial proteins, enzymes, electrolytes, and metabolites that collectively uphold ocular surface defense and stability (5). A meta-analysis confirmed a significant reduction in tear secretion among diabetic patients compared with non-diabetic controls. Subgroup analyses further verified this difference after adjusting for potential confounders (23). These findings suggest that, at early stages of the disease, diabetic dry eye may predominantly present as an aqueous-deficient subtype (64, 67).

In addition to reduced aqueous tear secretion, diabetic patients frequently exhibit evaporative dry eye secondary to meibomian gland dysfunction (70, 71). The lipid layer of the tear film is primarily secreted by the meibomian glands and functions to reduce tear evaporation and minimize ocular surface shear stress. Hyperglycemia-related metabolic disturbances and structural alterations of the glands can disrupt the integrity of the tear film lipid layer. *In vivo* laser scanning confocal microscopy studies have demonstrated cytological and compositional abnormalities in the meibum of patients with diabetes (72). Clinical studies have further reported increased meibomian gland dropout in diabetic patients compared with healthy controls, accompanied by reduced lipid layer thickness and decreased tear meniscus height (73, 74).

The mucin layer on the ocular surface consists of membrane-bound, secreted, and tethered mucins, which work synergistically to preserve a hydrated, lubricated, and pathogen-resistant barrier at the tear–epithelium interface (75). In diabetes, damage to the epithelial cells alters mucin organization and decreases its presence in the tear film, compromising tear stability and epithelial protection (76–78).

**Figure 2:**
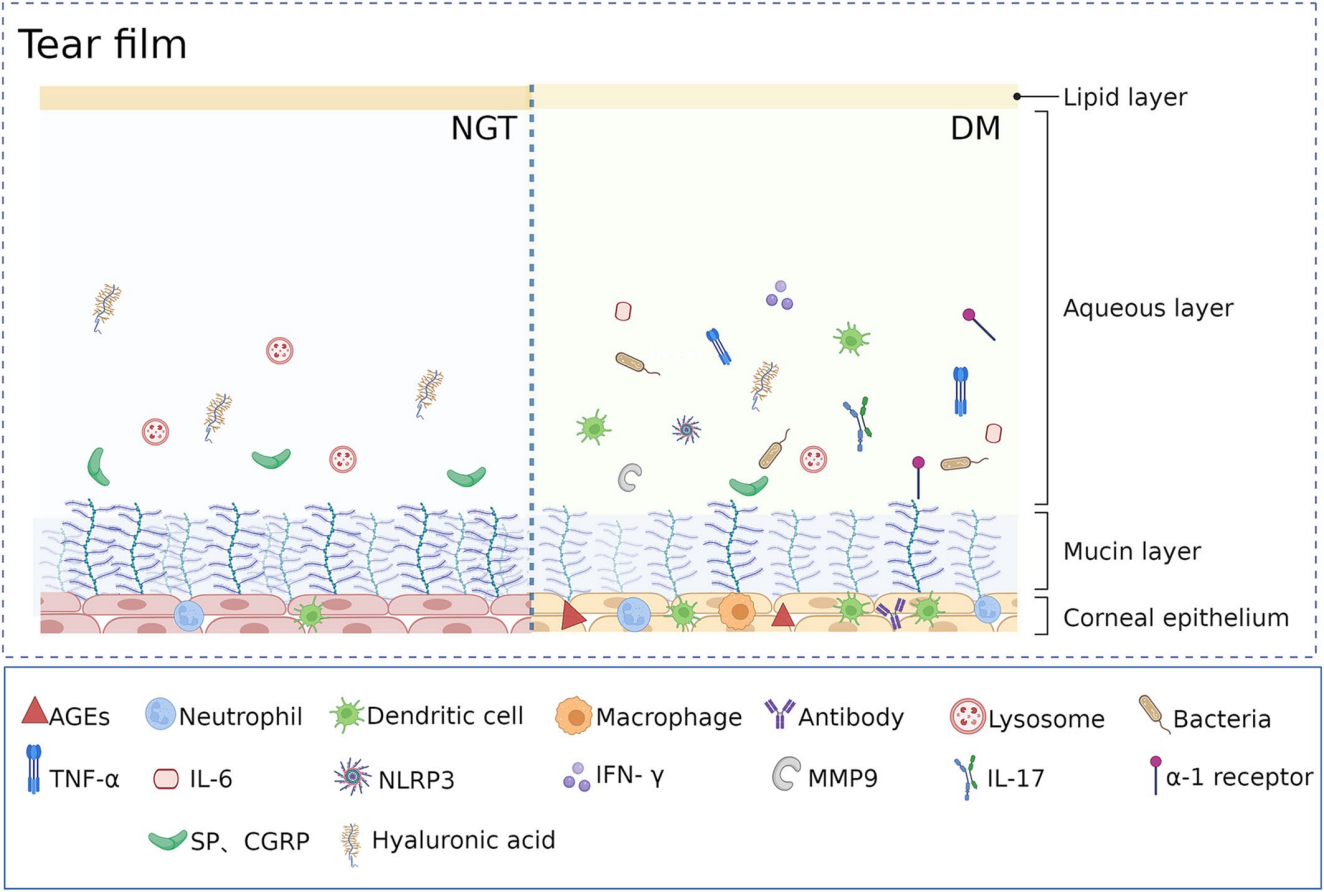
Comparison of tear film structure and composition between individuals with normal glucose tolerance and patients with diabetes. Created in BioRender. Ting, H. (2026) https://BioRender.com/cut04gk.

Beyond structural compromise of the aqueous, mucin, and lipid layers, diabetic tear fluid also shows elevated glucose levels and diminished lysosomal content (79). Dysfunction of the LFU increases the expression of immune cells, immunoglobulins, metabolic waste, and pro-inflammatory cytokines in the tear film, while reducing levels of neurotrophic factors (80–83). These compositional shifts likely contribute to the elevated tear osmolarity observed in diabetic dry eye. (Figure 2).

## 4 Diagnosis

### 4.1 Manifestations

A descriptive hospital-based study identified foreign body sensation and blurred vision as the most commonly reported complaints (84). Han et al. further reported that, in patients with diabetic dry eye, SPEED scores were significantly negatively correlated with corneal nerve density as well as the length and number of major nerve fibers. In contrast, non-invasive tear breakup time (NIBUT) and Schirmer I test (SIt) wetting length showed positive correlations with the density and number of major corneal nerve fibers (85). Overall, diabetic dry eye is associated with more severe symptoms and ocular surface damage than non-diabetic dry eye, in close relation to structural changes in corneal nerves (86–89). However, these non-specific ocular discomforts and routine clinical signs are often regarded as minor and consequently overlooked, thereby increasing the risk of underdiagnosis or delayed diagnosis of diabetic dry eye. Notably, as the disease progresses, severe corneal nerve damage may lead to a marked reduction in sensory input, such that subjective symptom perception can be attenuated even in the presence of significantly elevated tear osmolarity (90, 91). This phenomenon indicates that reliance on symptom-based questionnaires alone may be insufficient for the accurate identification of diabetic dry eye. Therefore, comprehensive evaluation strategies that incorporate the distinct pathophysiological features of diabetic dry eye are warranted in diabetic populations to enable more accurate diagnosis and timely intervention.

### 4.2 Diagnostic strategies

In addition to the common tear film and ocular surface abnormalities shared with other dry eye subtypes, corneal sensitivity should be regarded as a specific diagnostic indicator for diabetic dry eye. However, ocular tissue damage associated with DM may initiate as early as the impaired glucose tolerance stage (58, 92). A marked decline in corneal sensation typically signifies more advanced disease progression. Earlier and more timely differential diagnosis can be facilitated through the use of IVCM and tear biomarker analysis (45).

**Figure 3:**
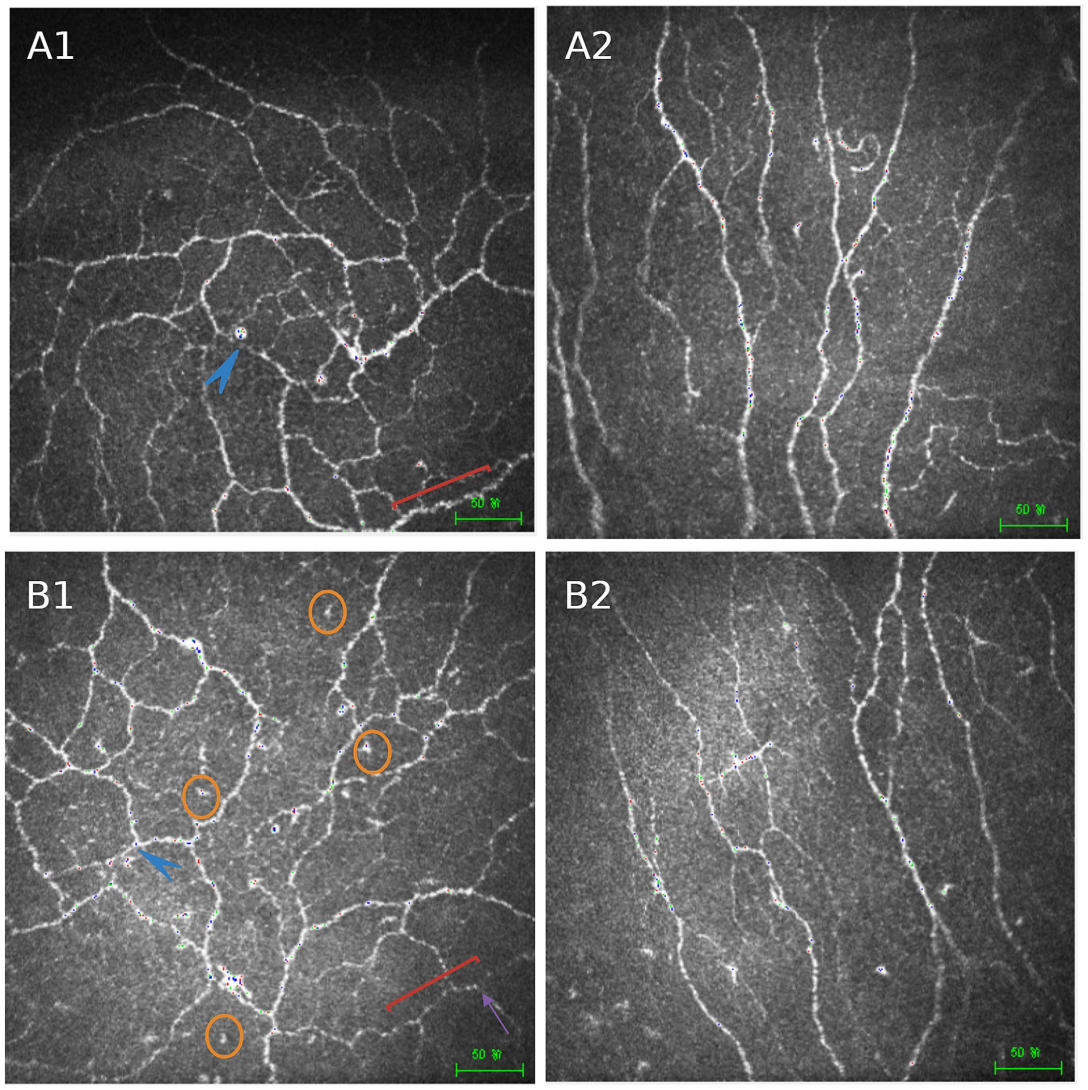
Comparison of corneal sub-basal nerve plexus morphology between normal individuals and diabetic patients. Representative IVCM images of the corneal sub-basal nerve plexus are shown. Panels **(A1,A2)** illustrate the central and peripheral corneal regions in normal individuals, respectively, demonstrating a well-organized inferior whorl pattern, thick nerve fibers, high nerve density, and abundant nerve branching. Panels **(B1,B2)** show the corresponding regions in patients with diabetes, characterized by structural disorganization of the inferior whorl region, reduced nerve fiber density, shorter and thinner nerve fibers, decreased branching, increased nerve fiber tortuosity, and fewer nerve beadings. Blue arrows indicate the inferior whorl region. Orange circles highlight infiltrating inflammatory cells. Red brackets and purple arrows denote nerve beadings, which are reduced in diabetic corneas. These morphological alterations reflect corneal nerve impairment associated with chronic hyperglycemia and are frequently observed in diabetic dry eye disease. Scale bars = 50 μm. Created in BioRender. Ting, H. (2026) https://BioRender.com/nvj04as.

IVCM enables real-time, high-resolution imaging of corneal microstructures, with particular emphasis on the inferior whorl region of the sub-basal nerve plexus (SNP). This site is widely recognized as a reliable location for assessing corneal nerve architecture and early neurodegenerative alterations (93, 94). Recent advances in wide-field imaging have further improved spatial resolution, contributing to a more refined understanding of diabetic corneal neuropathy (95). Despite its diagnostic utility, IVCM remains a contact-based technique. In patients with epithelial fragility, as frequently seen in diabetes, the procedure may increase the risk of epithelial complications, such as persistent defects or ulceration (57). In addition, its high cost and limited accessibility constrain broader clinical implementation, underscoring the need for more scalable and widely available diagnostic strategies (Figure 3).

Tear fluid contains a complex repertoire of biomolecules, providing a unique and non-invasive means to investigate ocular surface pathophysiology. The ease of collection, dynamic composition, and rich molecular content have established tear-based biomarkers as promising tools for precise diagnosis and management in a variety of clinical conditions (96). Tear samples collected via glass capillaries or Schirmer strips can be rapidly profiled using integrated multi-omics techniques to quantify cytokines, proteins, and microRNAs (80, 97). This approach exhibits high reliability in differentiating disease states (98). In diabetic dry eye, consistently altered tear levels of IL-6, IL-8, TNF-*α*, MMP-9, SP, neuropeptide Y, IGFBP-3, and progranulin have been reported, highlighting their potential as specific biomarkers (35, 36, 99–101). These insights support a move from broad-spectrum therapies toward personalized treatment strategies, which may redefine the management of ocular surface diseases.

In the early stages, diabetic dry eye may present with non-specific ocular discomfort, whereas at more advanced stages, it can manifest as a clinical pattern in which subjective symptoms are relatively attenuated despite progressive tissue damage, thereby increasing the risk of underdiagnosis or delayed diagnosis. Evidence from animal studies (102) suggests that ocular surface damage may be at least partially reversible when relevant pathological processes are appropriately modulated, highlighting the potential value of implementing early screening strategies in diabetic populations. Kalra et al. (103) were among the pioneers to propose integrating dry eye evaluations, as outlined in the TFOS DEWS II framework, into routine diabetes care. They emphasized that all patients with diabetes, particularly those with elevated HbA1c levels, DR, or DN, should undergo comprehensive ocular surface evaluations at least annually to enable the early detection of subclinical abnormalities. Diagnostic accuracy may be enhanced by incorporating corneal nerve imaging, tear-based biomarkers, and corneal sensitivity testing, thereby facilitating more personalized therapeutic strategies. However, this approach is still in its infancy, and standardized thresholds and grading criteria for these biomarkers remain to be established.

## 5 Treatment

Clinical management of dry eye typically follows expert consensus guidelines, focusing on treatments tailored to underlying etiologies and individualized according to disease subtype and severity (14). Optimal glycemic control rapidly enhances tear film stability; when combined with artificial tears, meibomian gland hygiene, and corneal epithelial barrier restoration, it can significantly alleviate symptoms (104–106). However, conventional dry eye therapies often fall short of addressing diabetes-specific pathophysiological mechanisms, which may partly account for the limited and recurrent clinical efficacy observed in diabetic patients. As insights into the mechanisms of diabetes-associated ocular surface damage continue to deepen, an increasing number of targeted therapeutic approaches have begun to emerge.

### 5.1 Baseline management and glycemic control

Clinical studies suggest that sodium–glucose cotransporter 2 (SGLT2) inhibitors and glucagon-like peptide-1 (GLP-1) receptor agonists may exert protective effects on the ocular surface, with their use being associated with a lower risk of dry eye. Specifically, SGLT2 inhibitors have been demonstrated to mitigate dry eye severity and reduce corneal dendritic cell density, whereas GLP-1 receptor agonists may lower the incidence of superficial punctate keratitis (107–110). Although the relationships between metformin or pioglitazone and dry eye warrant further elucidation, existing data indicate that metformin may improve corneal nerve parameters and modulate neuroimmune status, suggesting potential neuroprotective effects in diabetic patients (111). Moreover, pioglitazone and basal insulin therapy have shown efficacy in promoting corneal nerve regeneration (112). Collectively, these antidiabetic agents hold promise for maintaining ocular surface integrity and provide a basis for personalized glycemic control strategies in the management of diabetic dry eye.

### 5.2 Neurotrophic and neuromodulatory therapies

In a randomized, double-blind trial, the combination of topical citicoline and vitamin B12 significantly enhanced corneal sensitivity and alleviated ocular symptoms in diabetic patients (113). Nerve growth factor (NGF) has been demonstrated in both *in vivo* and *in vitro* studies to promote corneal epithelial repair and enhance tear secretion (114). Recombinant human NGF (rhNGF) has been approved for the treatment of neurotrophic keratitis, suggesting the potential applicability of neurotrophic strategies in corneal-related disorders (115). In addition, intranasal neurostimulation, as a device-based therapeutic approach, can improve tear secretion and tear film stability by activating lacrimal reflex pathways (116–118). However, evidence supporting its application in diabetic dry eye remains limited, and its clinical use in this context is still largely exploratory.

Recent insights from Professor Lixin Xie’s team indicate that sympathetic overactivation contributes to mitochondrial dysfunction in the lacrimal gland, with α1-adrenergic receptors playing a key role in tear secretion regulation (10). Preclinical studies reveal that α1-adrenergic antagonists improve tear production and mitigate meibomian gland and goblet cell dysfunction in diabetic models (65). These findings uncover a novel neuro-energetic pathway influencing tear secretion. Ongoing clinical trials led by Professor Xie aim to translate these discoveries into targeted therapies for diabetic dry eye.

### 5.3 Secretagogues

Diquafosol sodium is a P2Y₂ receptor agonist that promotes tear fluid and mucin secretion, thereby improving tear film stability (63). An exploratory study in patients with type 2 diabetic dry eye demonstrated that diquafosol sodium improved tear film parameters and alleviated clinical symptoms (119). Subsequent randomized controlled trials further indicated that 3% diquafosol sodium may offer advantages over sodium hyaluronate in enhancing tear film stability and increasing corneal nerve density (120). It should be noted that Diquafosol ophthalmic solution is not a direct anti-inflammatory or neurotrophic agent, and the observed improvements in corneal nerve parameters are more likely secondary to restoration of the ocular surface microenvironment and tear film homeostasis.

### 5.4 Anti-inflammatory therapy

Anti-inflammatory therapy remains a foundational strategy in managing diabetic dry eye, targeting both ocular surface injury and tear film instability. Conventional agents, such as cyclosporine, short-term corticosteroids, and non-steroidal anti-inflammatory drugs (NSAIDs), effectively suppress inflammatory cascades, thereby preserving goblet cell integrity and supporting tear film homeostasis. However, prolonged use of these agents may compromise corneal epithelial health and diminish corneal sensitivity, potentially limiting their long-term suitability in diabetic populations (121).

Topical ocular insulin has been shown to downregulate proinflammatory mediators, such as IL-1α, IL-6, and MMP-9, while concurrently improving symptoms and enhancing tear production in patients with diabetes (122). In addition, a composite formulation containing chondroitin sulfate and myo-inositol has been reported to improve corneal sensitivity, prolong tear breakup time, and reduce OSDI scores, potentially through modulation of the PI3K/AKT signaling pathway (123). However, the current evidence supporting these interventions remains limited.

### 5.5 Antioxidant therapy

Oxidative stress plays an important role in the initiation and progression of diabetic dry eye. In a randomized, placebo-controlled trial, 6 months of omega-3 fatty acid supplementation significantly increased corneal nerve fiber length in patients with type 1 diabetes mellitus (124). Similarly, clinical data indicate that co-administration of α-lipoic acid with hydroxypropyl methylcellulose (HPMC) results in greater improvements in TBUT, OSDI scores, tear film morphology, and corneal staining compared to HPMC alone (125). The therapeutic effects of α-lipoic acid are largely attributed to its potent antioxidative capacity, such as ROS scavenging, metal-ion chelation, and enhancement of endogenous antioxidant systems, ultimately promoting corneal nerve health in diabetic individuals (126). These findings highlight the potential of targeted antioxidant therapy to mitigate ocular surface damage and support neural repair in diabetic dry eye.

### 5.6 Emerging and preclinical strategies

Acupuncture is a traditional therapeutic modality with a history of more than two millennia. Neuroanatomical studies have confirmed that acupoints and meridians are densely innervated, and their stimulation triggers the release of neurotransmitters, neuropeptides, and immune mediators, thereby modulating sensory input, inflammation, and systemic balance (127, 128). Increasing evidence underscores acupuncture’s crucial role in neural repair, stimulating nerve regeneration and axonal sprouting through the activation of neurotrophic factors (129, 130). Acupuncture has demonstrated potential benefits in alleviating dry eye symptoms and improving tear film stability, and several meta-analyses indicate that acupuncture combined with artificial tears may be more effective than control treatments alone (131). However, high-quality clinical evidence specifically supporting its efficacy in diabetic dry eye remains limited.

Other therapeutic approaches, such as peroxisome proliferator-activated receptor gamma (PPARγ) agonists (132), probiotics (133), and modulation of the OGF–OGFR growth regulatory axis (102, 134), have exhibited potential in preclinical diabetic dry eye models. However, these findings predominantly originate from animal studies, and further well-designed clinical trials are necessary to confirm their safety and efficacy in humans.

## 6 Conclusion and future directions

Diabetic dry eye is highly prevalent, particularly among patients with suboptimal glycemic control. The pathogenesis of this condition is multifactorial, primarily driven by glucose-induced corneal neuropathy, which results in dysfunction of the LFU, decreased tear production, and altered tear composition. These pathological processes often lack specific symptoms in the early stages of the disease, but can present as a clinical phenotype characterized by “relatively mild symptoms but significant tissue damage” during the progression stage. This highlights the importance of conducting regular ocular surface screening in the diabetic population.

In clinical practice, the early identification rate of diabetic dry eye should be improved by combining corneal sensation assessment, *in vivo* confocal microscopy examination based on the inferior whorl region of the SNP, and the detection of tear fluid biomarkers (such as IL-6, MMP-9, and P substance). While current therapies primarily focus on symptom alleviation, emerging treatments targeting underlying mechanisms, such as glycemic control, neuroregulation, anti-inflammation, and antioxidation, have shown promising outcomes. Further high-quality randomized controlled trials are required to better define the optimal target populations, dosing regimens, and long-term efficacy of these interventions.

Currently, diabetic dry eye lacks universally accepted diagnostic criteria and is predominantly diagnosed based on clinical features. This absence of standardized definitions complicates patient characterization and limits the clarity of therapeutic outcomes in clinical trials. Establishing consensus on disease nomenclature and quantifiable diagnostic benchmarks will enhance the precision of study results and clarify the relationship between disease pathophysiology and treatment mechanisms in randomized controlled trials. Future efforts should prioritize the standardization of clinical definitions and diagnostic protocols for diabetic dry eye, aligned with current diabetes management guidelines, to support more reliable data integration and comparability across studies.
